# Volatile compound-mediated plant–plant interactions under stress with the tea plant as a model

**DOI:** 10.1093/hr/uhad143

**Published:** 2023-07-23

**Authors:** Jieyang Jin, Mingyue Zhao, Tingting Jing, Mengting Zhang, Mengqian Lu, Guomeng Yu, Jingming Wang, Danyang Guo, Yuting Pan, Timothy D Hoffmann, Wilfried Schwab, Chuankui Song

**Affiliations:** State Key Laboratory of Tea Plant Biology and Utilization, International Joint Laboratory on Tea Chemistry and Health Effects, Anhui Agricultural University, 230036, Hefei, Anhui, China; State Key Laboratory of Tea Plant Biology and Utilization, International Joint Laboratory on Tea Chemistry and Health Effects, Anhui Agricultural University, 230036, Hefei, Anhui, China; State Key Laboratory of Tea Plant Biology and Utilization, International Joint Laboratory on Tea Chemistry and Health Effects, Anhui Agricultural University, 230036, Hefei, Anhui, China; State Key Laboratory of Tea Plant Biology and Utilization, International Joint Laboratory on Tea Chemistry and Health Effects, Anhui Agricultural University, 230036, Hefei, Anhui, China; State Key Laboratory of Tea Plant Biology and Utilization, International Joint Laboratory on Tea Chemistry and Health Effects, Anhui Agricultural University, 230036, Hefei, Anhui, China; State Key Laboratory of Tea Plant Biology and Utilization, International Joint Laboratory on Tea Chemistry and Health Effects, Anhui Agricultural University, 230036, Hefei, Anhui, China; State Key Laboratory of Tea Plant Biology and Utilization, International Joint Laboratory on Tea Chemistry and Health Effects, Anhui Agricultural University, 230036, Hefei, Anhui, China; State Key Laboratory of Tea Plant Biology and Utilization, International Joint Laboratory on Tea Chemistry and Health Effects, Anhui Agricultural University, 230036, Hefei, Anhui, China; State Key Laboratory of Tea Plant Biology and Utilization, International Joint Laboratory on Tea Chemistry and Health Effects, Anhui Agricultural University, 230036, Hefei, Anhui, China; Biotechnology of Natural Products, Technische Universität München, Liesel-Beckmann-Str. 1, 85354 Freising, Germany; Biotechnology of Natural Products, Technische Universität München, Liesel-Beckmann-Str. 1, 85354 Freising, Germany; State Key Laboratory of Tea Plant Biology and Utilization, International Joint Laboratory on Tea Chemistry and Health Effects, Anhui Agricultural University, 230036, Hefei, Anhui, China

## Abstract

Plants respond to environmental stimuli via the release of volatile organic compounds (VOCs), and neighboring plants constantly monitor and respond to these VOCs with great sensitivity and discrimination. This sensing can trigger increased plant fitness and reduce future plant damage through the priming of their own defenses. The defense mechanism in neighboring plants can either be induced by activation of the regulatory or transcriptional machinery, or it can be delayed by the absorption and storage of VOCs for the generation of an appropriate response later. Despite much research, many key questions remain on the role of VOCs in interplant communication and plant fitness. Here we review recent research on the VOCs induced by biotic (i.e. insects and pathogens) and abiotic (i.e. cold, drought, and salt) stresses, and elucidate the biosynthesis of stress-induced VOCs in tea plants. Our focus is on the role of stress-induced VOCs in complex ecological environments. Particularly, the roles of VOCs under abiotic stress are highlighted. Finally, we discuss pertinent questions and future research directions for advancing our understanding of plant interactions via VOCs.

## Introduction

Plants produce and release a large number of volatiles, especially terpenoids, which are the dominant class of secondary metabolites released in response to external stimuli [1–3]. These low-molecular-weight chemicals are dispersed in the air at ambient temperatures and play diverse roles in plants [4]. To date, more than 1000 different plant-emitted volatile organic compounds (VOCs) have been identified [5–7]. Floral volatiles are frequently released continuously and can act as attractants for pollinators and as defensive mechanisms against florivores [8]. Additionally, they can facilitate interactions with other organisms [9]. While vegetative plant volatiles were previously regarded as mere metabolic byproducts, a growing amount of research indicates that they play various roles in the plant lifespan through direct and indirect defense mechanisms, as well as in plant interactions with other organisms and the surrounding environment [9–12]. To date, over 30 species have been shown to engage in plant–plant communication through VOCs [6]. Neighboring plants use VOCs to compete for scarce resources and bolster defenses against stresses [13, 14]. The effectiveness of volatile communication between plants depends on the context and is contingent on the particular interacting species. Given that neighboring plants face different types of stress, it is essential for plants to detect and respond to their neighbors, regardless of how the interaction ultimately plays out. When a plant is exposed to specific stressors, it releases a unique blend of volatiles that can activate or suppress pathways in neighboring plants to prepare for subsequent stress.

Tea plants (*Camellia sinensis*) are among the most important economic crops in China and other tea-producing countries, such as India, Kenya, and Japan. Tea plants contains a rich variety of volatile compounds, and these compounds have major effects on tea quality and plant performance [15, 16]. In contrast to model plants, tea plants can produce and emit abundant VOCs (>700 volatiles) during their growth and development, and these VOCs affect the performance and quality of tea plants [16]. Therefore, tea plants should be better suited for analyses of the VOCs that mediate plant–plant interactions than *Arabidopsis*, which only produces trace amounts of volatiles.

The biosynthesis and role of volatiles in plant–plant communication have garnered increasing research attention. Biotic stresses, such as pathogen attacks and tea green leafhopper infestation, as well as abiotic stresses like extreme temperature and wounding, can lead to the accumulation of distinct aroma compounds in tea. In this review we examine the volatiles released by tea plants in response to both biotic and abiotic stress, their impact on biosynthesis pathways, and their functions, with a specific focus on the volatiles generated under abiotic stress. We conclude this review with a brief discussion of future directions for research that could broaden our comprehension of the functions of VOCs in plant interactions.

## Volatiles induced by abiotic and biotic stress in tea plants

**Figure 1:**
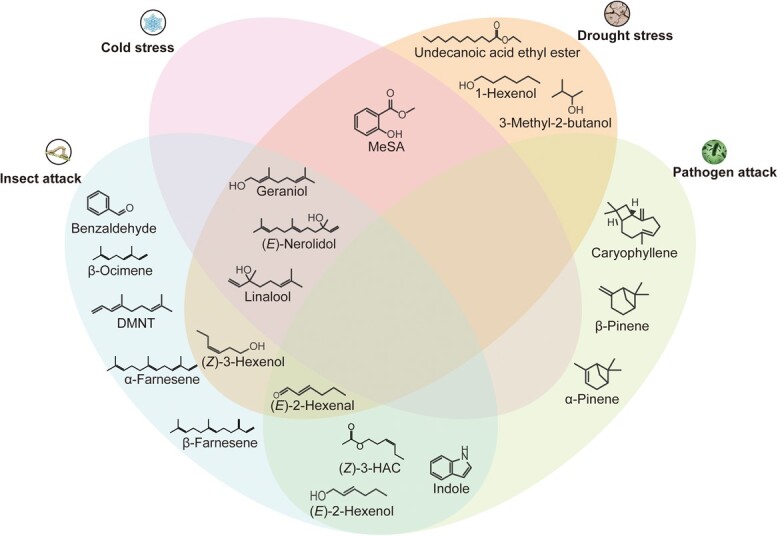
VOCs released from plants under different stresses.

Tea plants experience varying degrees of damage under various types of environmental stress (Fig. 1). The growth and quality of tea plants can be significantly compromised by various abiotic factors, with low temperature, high salt levels, and drought being the most prominent culprits. Additionally, diseases and pests can also have adverse impacts on tea plant metabolism, further undermining their overall performance.

The volatiles induced by different stresses in tea plants have received increased research attention [17–22] (Fig. 1). While the emission of monoterpenoids is triggered by all types of stress, the production of aromatic volatiles such as benzaldehyde appears to be promoted only by insect attack. Fatty acid degradation products such as (*E*)-2-hexanal and (*Z*)-3-hexenol are again apparently general response signals, since they are triggered by many stress factors.

## Biosynthesis of stress-related volatiles in tea plants

**Figure 2:**
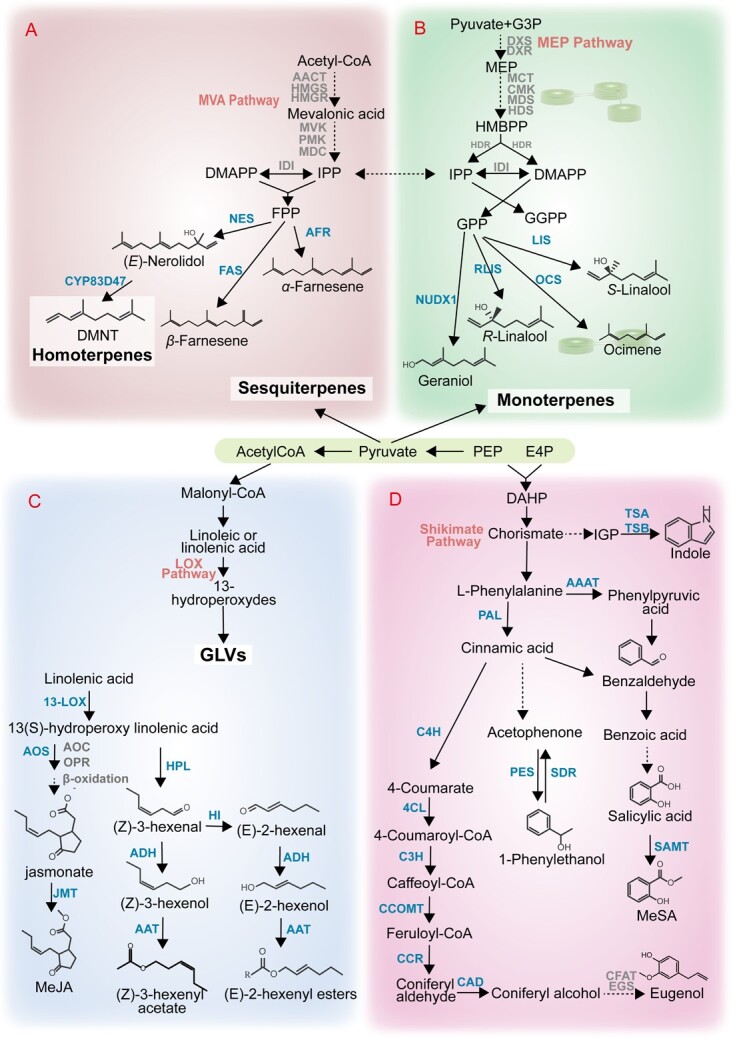
Biosynthetic pathways of stress-induced volatiles in the tea plant. (A, B) Biosynthesis of stress-induced volatile terpenes. MEP, methylerythritol phosphate; MVA, mevalonic acid; DMAPP, dimethylallyl pyrophosphate; IPP, isopentenylpyrophosphate; FPP, farnesyl diphosphate; FAS, β-farnesene synthase; AFR, α-farnesene synthase; LIS, *S*-linalool synthase; RLIS, *R*-linalool synthase; OCS, β-ocimene synthase; NES, nerolidol synthase; NUDX1, nudix hydrolase 1. (C) Biosynthesis of stress-induced volatiles from the fatty acid degradation pathway. LOX, lipoxygenase; AOS, allene oxide synthase; ADH, alcohol dehydrogenases; JMT, jasmonic acid carboxyl methyltransferase; HI, (*Z*)-3:(*E*)-2-hexenal isomerase; AAT, alcohol acetyltransferases. (D) Biosynthesis of stress-induced volatiles from phenylpropanoid/benzenoid pathway, TSA, tryptophan synthase α-subunit; TSB, tryptophan synthase β-subunits; AAATs, aromatic amino acid aminotransferases; PAL, phenylalanine ammonia lyase; PES, 1-phenylethanol synthase; SDR, short-chain dehydrogenase/reductase; SAMT, salicylic acid carboxyl methyltransferase; IGP, indole-3-glycerol phosphate; C4H, cinnamate 4-hydroxylase; CCOMT, caffeoyl-CoA O-methyltransferase; CCR, cinnamoyl-CoA/coniferyl-CoA reductase; CAD, cinnamyl alcohol dehydrogenase; CFAT, coniferyl alcohol acyl transferases; EGS, eugenol synthase. PEP, phosphoenolpyruvate; E4P, erythrose 4-phosphate. Blue characters indicate known genes in tea. Dotted arrows: unclear step.

Stress-induced volatiles can be broadly categorized into four major classes based on their metabolic origin, including degradation products of volatile fatty acids, such as insect herbivore-induced (*Z*)-3-hexenol and (*Z*)-3-hexenyl acetate; volatile phenylpropanoids/benzenoids, such as cold or drought stress-induced methyl salicylate (MeSA); volatile monoterpenes, such as pinenes, linalool and ocimene triggered by biotic and abiotic stress; and sesquiterpenes, such as cold stress-induced nerolidol (Fig. 2).

### Stress-related volatiles from the terpene pathway

Terpenoids represent the most diverse class of plant secondary metabolites (Fig. 2A and B). More than 100 terpene synthases (TPSs) have been identified to date [23], although only a few *TPS* genes have been characterized in tea plants. Linalool is synthesized by linalool synthase (CsLIS1 and CsLIS2), and mechanical damage caused by *Empoasca onukii* attack is a key factor affecting linalool accumulation and emission in tea plants [17]. The two enantiomers of linalool, (*R*)- and (*S*)-linalool, are present in tea leaves. The (*R*)-linalool synthase gene is stress-responsive and induces the accumulation of (*R*)-linalool during oolong tea processing [24]. The two splicing forms of *CsLIS/NES* encode nerolidol synthase (NES) and linalool synthase (LIS), which are responsible for the production of (*E*)-nerolidol and linalool *in planta* [25]. One previous study has analyzed the emission pattern of *β*-ocimene triggered by tea geometrids and the mechanism that regulates its emission [26]. The TPS related to the synthesis of *β*-ocimene and *β*-farnesene has been cloned from tea plants [27]. *β*-Farnesene serves as a critical and commonly conserved alarm pheromone in the presence of aphids. Two sesquiterpene synthases, CsAFR and CsNES2, are induced by herbivory and are responsible for the increased emission of *α*-farnesene and (*E*)-nerolidol [28]. CsAFR only participates in *α*-farnesene formation, and the emission of α-farnesene may act as a signal to induce antibacterial-related responses in adjacent undamaged tea leaves [29]. Recently it has been discovered that the P450 enzyme CsCYP82D47 facilitates the final step of DMNT biosynthesis from (*E*)-nerolidol in tea plants [30] (Fig. 2A and B).

### Stress-related volatiles from the fatty acid degradation pathway

Volatile fatty-acid derivatives are important secondary metabolites in tea plants that significantly contribute to the aroma of tea products (Fig. 2C) [31, 32]. These stress-induced volatiles include (*Z*)-3-hexenal, (*Z*)-3-hexenol, and (*Z*)-3-hexenyl esters, as well as methyl jasmonate (MeJA), which are produced from C18 unsaturated fatty acids [33]. The unsaturated fatty acids are subjected to stereospecific oxygenation, resulting in the formation of 13-hydroperoxy intermediates, which are then metabolized to produce volatile C6 compounds [34–36]. (*E*)-2-Hexenal also plays a critical role in response to biotic/abiotic stresses, and recently a new (*Z*)-3:(*E*)-2-hexenal isomerase (CsHI) was identified in tea plants; CsHI catalyzes the conversion of (*Z*)-3-hexenal to (*E*)-2-hexenal [37]. Aldehydes can undergo further metabolism by alcohol dehydrogenases (ADHs) [38, 39] to form their corresponding alcohols, and these alcohols can be converted to esters catalyzed by alcohol acyl-CoA transferases (AATs). However, the specific AATs involved in tea plants remain unknown. Additionally, apart from aldehydes and alcohols, jasmonic acid (JA) and MeJA are important fatty acid derivatives. MeJA is synthesized from 13S-hydroperoxylinolenic acid through a pathway that involves 12,13-epoxylinolenic acid and JA. The allene oxide synthase 2 (AOS2) gene is the only gene involved in JA synthesis and is expressed in tea flowers following insect attack [40]. Finally, JA can be methylated into MeJA by jasmonate carboxyl methyltransferase [41] (Fig. 2C).

### Stress-related volatiles from the l-phenylalanine pathway

Volatile phenylpropanoid/benzenoid (VPB) compounds (benzaldehyde, benzyl alcohol, 2-phenylethanol, eugenol, and indole) are the second most ubiquitous class of plant volatiles [42], and they mainly serve as signals for pollinator attraction (Fig. 2D) [43]. The majority of volatiles in this category are produced from l-phenylalanine, which is derived from shikimate. The well-known precursor pathway for VPB formation involves the catalysis of l-phenylalanine to *trans*-cinnamic acid by l-phenylalanine ammonia lyase (PAL) enzymes [44, 45]. Although volatile terpenes and fatty acid derivatives are commonly implicated in plant interactions with the environment, VPBs are primarily present in floral tissues of plants [43]. For example, 2-phenylethanol is found in rose flowers [46] and 1-phenylethanol (1PE) is found in tea flowers [47]. In tea flowers, the 1PE synthase CsPES has the highest catalytic efficiency for the conversion of acetophenone [48]. Moreover, the activities of tea leaves containing such enzymes were equivalent to those of flowers [47]. MeSA, which is generated from salicylic acid (SA) by the enzyme salicylic acid carboxyl methyl transferase (SAMT), plays a significant role in plant defense mechanisms. In the withering process of white tea production, MeSA is produced, resulting in the formation of a distinct floral scent [49]. Indole is a prevalent component of *C. sinensis* and plays a crucial role in tea plants. It is synthesized in tea leaves from indole-3-glycerol phosphate (IGP) by tryptophan synthase α-subunit (TSA), and the activity of CsTSA is primarily reliant on its interaction with the tryptophan synthase β-subunit [50]. Eugenol, a floral volatile compound, is a significant constituent in numerous aromatic plants [51]. The pathway leading to eugenol formation is complex and partly elucidated [52–55]. The final enzymes of this pathway, alcohol acyl transferases (CFAT) and eugenol synthase (EGS), are still unknown in the tea plant (Fig. 2D).

## Roles of volatiles in plant–plant interactions under biotic stress

### Herbivore attack

**Figure 3:**
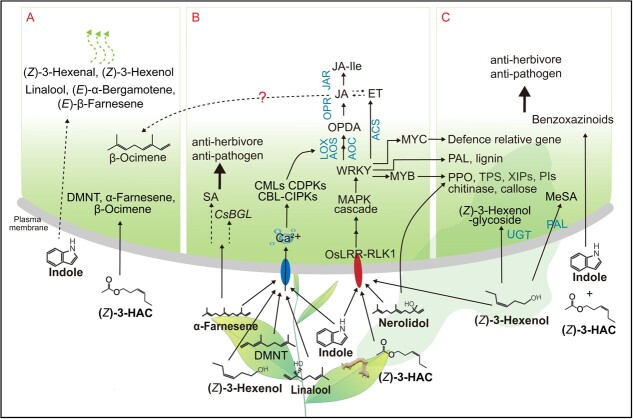
Biological function of VOCs under herbivore attack. (A) Insect-induced VOCs trigger neighboring plants to release other blends of volatiles. (B) Insect-induced VOCs perform defensive functions against insects via Ca^2+^ signaling, MAPK, LOX, JA, and SA pathways. (C) Insect-induced VOCs absorbed and transformed into bioactive compounds. CsBGL, β-1,3-glucanase; Rboh, NADPH oxidase/respiratory burst oxidase homolog; LOX, lipoxygenase; AOS, allene oxide synthase; AOC, allene oxidecyclase; JAR, jasmonate resistance; JA, jasmonic acid; SA, salicylic acid; MAPK, mitogen-activated protein kinase; ACS, aminocyclopropane carboxylic acid synthase; ET, ethylene; PAL, phenylalanine ammonia lyase; PES, 1-phenylethanol synthase; PPO, polyphenol oxidases; TPS, terpene synthase; XIP, xylanase inhibitor proteins; PIs, proteinase inhibitors; UGT, UDP-glycosyl-transferase; Blue characters indicate known genes in tea. Dotted arrow with red question mark indicates an unclear step.

Induced by herbivores, indole enhances the emission of defensive volatiles in maize plants growing nearby, such as (*Z*)-3-hexenal, (*Z*)-3-hexenol, linalool, and (*E*)-β-farnesene [56] (Fig. 3A). Similarly, (*Z*)-3-hexenyl acetate (HAC) is released in gypsy moth-infested hybrid poplar, which can prime volatile DMNT, β-ocimene, and α-farnesene production [57] (Fig. 3A). In maize and hybrid poplar, HAC acts as a priming agent for JA production and enhances the emission of VOCs, as reported in previous studies [57, 58]. Furthermore, volatiles induced by *Tetranychus urticae* are distinct and show a different chemical profile compared with those induced by mechanical wounding, and their neighboring plants can recognize such leaf volatiles [59]. Some aroma signals need to be superimposed with other inducing factors to activate the specific resistance mechanism, and it is not clear how aroma signals and insect pest inducing signals superimpose to jointly activate the defense mechanism. Experiments conducted on individual volatile compound exposure demonstrated that insects induced the release of (*Z*)-3-hexenol, linalool, α-farnesene, and DMNT, which in turn triggered the emission of β-ocimene in adjacent undamaged tea plants. This response was found to be dependent on the Ca^2+^ signaling pathways as well as the JA signaling pathways involving, lipoxygenase 1 (LOX1), allene oxidecyclase (AOC), and allene oxide synthase (AOS). Consequently, the presence of β-ocimene acted as a potent repellent for mated females of *Ectropis obliqua* [60] (Fig. 3A and B). It has been reported that JAs can regulate the release of herbivore-induced plant volatiles [61, 62], and signaling interactions between JA and ethylene have been demonstrated to result in higher levels of volatile emission [60] (Fig. 3B). However, the relationship between endogenous JA and ethylene levels in the leaves and their trigger to release plant volatiles remains unclear. Pest-induced aromas further stimulate neighboring plants to release other blends of volatiles; whether these aromatic substances return to the emitters remains to be studied.

Terpenes have direct defensive functions against insects [63], and it has recently been shown that they act as signals for plant–plant interaction [59]. Insect-induced DMNT triggers the upregulation of *lipoxygenase 1* (*LOX1*), *LOX3*, and proteinase inhibitors (*PI*s), which enhance the resistance of adjacent healthy tea plants to insect attack [30] (Fig. 3B). α-Farnesene can increase the expression of *β-1,3-glucanase* (*CsBGL*) and the level of SA, and thus the resistance of undamaged tea leaves to bacteria [29] (Fig. 3B). The release of indole and (*E*)-nerolidol in tea plants triggered by insect attack can effectively activate the mitogen-activated protein kinase (MAPK) and WRKY pathways, leading to an increase in the production of JA, H_2_O_2_, and abscisic acid (ABA) [19, 64] (Fig. 3B). Moreover, indole triggers the upregulation of expression of *calmodulin-like protein*s (*CML*s), *calcium-dependent protein kinase*s (*CDPK*s), and *calcineurin B-like protein-interacting protein kinase*s (*CIPK*s), while it does not affect the expression of *calmodulin* (*CAM*), suggesting that indole priming is achieved upstream of Ca^2+^ signaling rather than through the Ca^2+^ binding process itself [64] (Fig. 3B). The release of indole induced by armyworm attack can enhance the resistance of rice plants to *Spodoptera frugiperda* [65]. In maize, indole exposure markedly increases the content of JA, JA-IIe, and ABA, as well as the transcription of JA-responsive genes [56]. The expression of nearly all of the well-studied JA biosynthesis genes is increased by indole after herbivore attack [64, 66, 67] (Fig. 3B). Unlike terpenes, indole, and MeSA, which are released hours after wounding, green leaf volatiles (GLVs) are released almost immediately after wounding; therefore, they are considered typical wound signals [58]. Insect-induced (*Z*)-3-hexenol can activate Ca^2+^-related genes, MAPK pathways, F-box genes, and receptor-like kinases [68, 69] (Fig. 3B). In tea plants, the roles of (*Z*)-3-hexenol, α-farnesene, β-ocimene, nerolidol[19], linalool, DMNT, and indole in defense against insect pests have been elucidated, and most of them could mediate the defense response of neighboring undamaged plants by activating the JA pathway [70].

VOCs can also act as priming agents through a mechanism involving their absorption and transformation into bioactive compounds (Fig. 3C). This mechanism includes the enzymes polyphenol oxidase, PAL, and chitinase and the biopolymers lignin and callose, which have extensive anti-herbivore or anti-pathogen effects [19, 64]. (*Z*)-3-Hexenol can be taken up and metabolized into glycosides in both tomato [71] and tea plants [72] (Fig. 3C). The glycosides may serve as both direct defensive compounds and indirect defense mechanisms [73]. Additionally, HAC and indole work together to regulate the biosynthesis of benzoxazinoids, which are crucial secondary metabolites that provide a strong response to herbivore attack and protect cereals against herbivores [69, 74] (Fig. 3C). However, pre-exposure to HAC and indole separately does not have a significant impact on the production of benzoxazinoids.

Numerous research studies have elucidated the function of terpene volatiles in attracting herbivore predators as a form of indirect plant defense. For instance, DMNT serves as an attractant for the predatory mite *Phytoseiulus persimilis* [75], while linalool and MeSA act as attractants for females of *Amblyseius potentillae* [76]. Airborne signals released from clipped sagebrush can increase the resistance of tobacco to herbivores [77], suggesting that there are interactions between species.

### Pathogen attack

**Figure 4:**
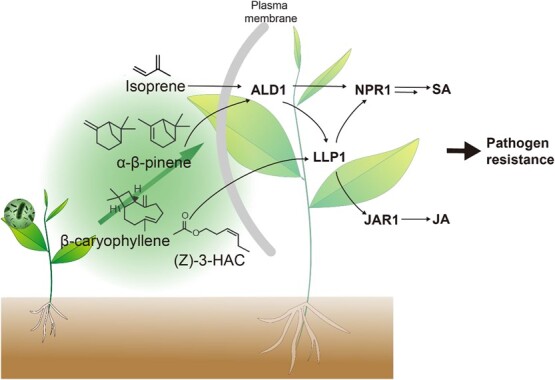
Biological function of VOCs under pathogen attack. ALD, AGD2-like defense response protein 1; NPR1, non-expressor of pathogenesis-related genes 1; LLP1, legume lectin like protein 1; JAR1, jasmonate resistance 1.

Few studies have examined priming by GLVs in plant–pathogen interactions [78, 79]. Exposure to certain VOCs confers resistance to the necrotrophic fungus *Botrytis cinerea* in *Arabidopsis thaliana* [80]. The beneficial effects of incorporating *Allium tuberosum* in intercropping systems on the resistance of banana (*Musa* spp.) to the necrotrophic fungal pathogen *Fusarium oxysporum* have been linked to the emission of VOCs [81]. Infected *Arabidopsis* plants emit α-pinene and β-pinene, which induce defense responses in neighboring plants [82] (Fig. 4). Thus, VOCs likely also enhance the resistance of plants to various types of pathogens.

Plants respond to pathogen infection by activating several layers of defense systems, including the production of defensive phytohormones, reactive oxygen species (ROS), specialized metabolites such as phytoalexins, physical barriers, and the expression of defense-related genes. These defense responses can be categorized into systemic acquired resistance (SAR) and induced systemic resistance (ISR) [83]. SAR is activated by salicylic acid (SA) in response to biotrophic pathogen infection, whereas ISR is mediated by JA and ethylene and enhances resistance to necrotrophic pathogen invasion [84]. Recent research suggests that VOCs can induce resistance to pathogens through different mechanisms, such as the priming of resistance gene expression in the receiver or direct inhibitory effects on microbial pathogens, resulting in a passive ‘associational’ resistance in the VOC-exposed plant [84].

The signaling pathways mediated by JA and SA trigger different defensive responses in plants, with SA signaling suppressing JA-mediated defenses [85, 86]. SA treatment in lima bean results in increased emissions of nonanal, which neighboring plants perceive and which induces the expression of *Pathogenesis-related protein*-2 (PR-2), thereby promoting resistance to *Pseudomonas syringae* infection [87]. However, this does not induce an indirect defense mechanism against herbivores in lima bean, but rather enhances resistance to bacterial pathogens. Studies using various *Arabidopsis* mutants deficient in either JA or SA signaling showed that isoprene activates SA-associated plant defense, while β-caryophyllene enhances the resistance of neighboring plants against *P. syringae* attack via JA-associated signaling [88] (Fig. 4). β-Caryophyllene binds to the transcriptional co-repressor TOPLESS complex, which modulates JA-mediated signaling [89].

(*Z*)-3-Hexenyl acetate [(*Z*)-3-HAC] enhances wheat resistance against *Fusarium* head blight by promoting JA-dependent defenses during the necrotrophic phase of *F. graminearum* infection, but suppresses SA-regulated defense during its biotrophic phase [90]. Prior exposure to (*Z*)-3-HAC leads to increased content of JA and 12-oxo-phytodienoic acid (12-OPDA) after caterpillar regurgitant application [91, 92], and thus confers higher resistance against insects and pathogens that are susceptible to JA-related plant defenses (Fig. 4).

Tea plants thrive in warm, humid climates with well-distributed rainfall and long hours of sunshine. However, monoculture conditions can create a stable microclimate that promotes the transmission and establishment of pathogens, as well as the growth of pests and diseases [93]. To date, 400 pathogens have been reported to infect tea plants; fungi comprise the largest proportion, followed by bacteria, viruses, and algae. The presence of blister blight disease has been shown to reduce the content of SA and JA in tea plant samples [94]. Despite this, little is known about the physiological function of pathogen-induced volatile compounds in tea plants. The SA and JA pathways are important for plant protection against pathogens and insects [95–99], emphasizing the need for studies on plant–microbe–insect interactions, which have increased in importance over the past two decades [100].

Our current understanding of how the identity of attackers, their order of arrival, and the biotic context influence the outcomes of interactions between plants, pathogens, and insects is restricted. Ponzio *et al*.’s review in 2013 emphasized the significance of considering both plant–microbe and plant–insect interactions in studies [100, 101]. While the effects of individual herbivores or microbes on volatile emissions have been extensively studied, we still have a limited understanding of how volatile emissions change when plants are attacked by multiple herbivores or microbes.

## Roles of volatiles in plant–plant interactions under abiotic stress

### Salt and drought stress

Tea plants prefer warm temperatures; cold temperatures and acidic and alkaline conditions are abiotic stresses that can have major effects on tea plants. Under salt stress, soil pH increases, which affects the absorption and transport of mineral elements by tea roots. Although numerous studies have characterized changes in volatile emissions by plants under biotic stress and examined the role of plant–plant communication in plant–herbivore interactions, few studies have examined these processes under abiotic stresses. Lee and Seo (2014) conducted a plant–plant communication experiment and found that neighboring receiver plants exhibited stress tolerance when exposed to VOCs emitted by salt-stressed *A. thaliana* [102]; likewise, non-stressed *Vicia faba* plants have the ability to perceive stress signals released by neighboring plants under salt stress and activate a priming response that could facilitate their response to subsequent salt stress [103]. Recently it has been revealed that volatiles from salt-stressed *Ocimum basilicum* plants increase the reproductive success of receivers under salinity [104].

Matthias Erb indicated that stomatal closure can be elicited by salt and drought stress and has been shown to reduce volatile emissions in maize [105], and it is believed that a reduction in constitutive volatile emissions may be indicative of a stressed neighbor. Under salt stress, this response is related to inhibition rather than the induction of volatile emission from the salt-stressed neighbors [105]. The prevalence and ecological importance of these silent volatile emissions have long been unclear [105].

**Figure 5:**
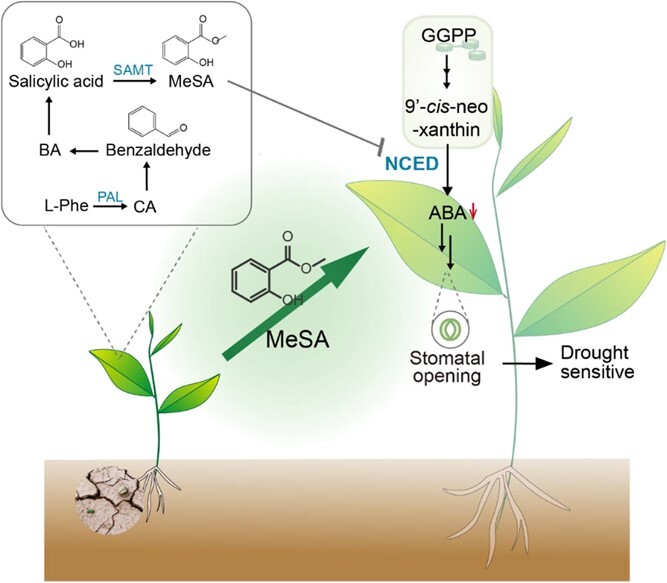
Biological function of VOCs under drought stress. Phe, phenylalanine; CA, cinnamic acid; PAL, phenylalanine ammonialyase; BA, benzoic acid; SAMT, salicylic acid carboxyl methyltransferase; GGPP, geranylgeranyl pyrophosphate; NCED, 9-*cis*-epoxycarotenoid dioxygenase.

The limited attention given to interactions between plants under drought stress has resulted in relatively little research. Nonetheless, some studies have reported that drought stress can modulate volatile emissions. For instance, in white spruce (*Picea glauca*) water deficit led to a significant reduction of 70% in photosynthesis, while only causing a decrease of 37% in the metabolic flux through the methylerythritol 4-phosphate (MEP) pathway, which governs isoprenoid biosynthesis [106]. In Scots pine seedlings, water deficit resulted in a decrease in the emission of certain sesquiterpenes, but had no impact on isoprene, monoterpenes, and oxygenated compounds [107]. In tea plants, hyperosmotic treatment leads to the accumulation of only a few compounds, such as (*Z*)-3-hexenol, (*E*)-2-hexenal, and MeSA; (*Z*)-3-hexenol application has been found to improve the hyperosmotic stress tolerance of tea plants, while pretreatments with (*E*)-2-hexenal and MeSA do not have the same effect [108]. In general, VOCs are known to play a positive role in regulating plant defense mechanisms. However, our recent findings suggest that volatiles induced by drought can actually decrease early drought tolerance in nearby plants [20]. Specifically, the presence of MeSA, which is induced by drought, can lower the ABA content in neighboring plants by reducing the expression of the *9-cis-epoxycarotenoid dioxygenase* (*NCED*) gene. This leads to inhibition of stomatal closure and the early drought tolerance reduction in adjacent plants (Fig. 5). The impact of MeSA is similar to that of cytokines and auxin; physiological concentrations of these hormones promote stomatal opening when their levels are low, but hinder it when their levels are high [109, 110]. It is not clear whether drought-induced volatiles inhibit drought resistance, or whether drought-induced reductions in volatiles contribute to the inhibition of drought resistance. Further investigation is necessary to comprehend the prevalence and identity of volatile compounds that suppress plant defense.

### Cold stress

Low temperature is a crucial environmental factor that limits the growth, survival, and geographical distribution of tea plants [108]. The (ICE1)-C-repeat-binding factor/dehydration-responsive element-binding factor (CBF/DREB)-cold-regulated gene (*COR*) pathway is the most extensively studied signaling pathway that regulates plant response to cold stress. The transcriptional cascade plays a vital role in cold response and has been detected in various plant species, including tea plants [111]. Additionally, Zhao *et al*. performed a genome-wide investigation of tea WRKY transcription factor genes, and the functional roles of two CsWRKY genes (*CsWRKY29* and *CsWRKY37*) in cold tolerance were fully validated [112].

**Figure 6:**
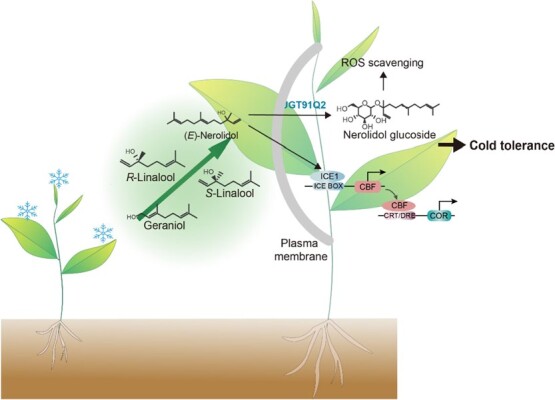
Biological function of VOCs under cold stress. ICE1, CBF expression 1; CBF, C-repeat-binding factor; COR, cold-regulated gene; ROS, reactive oxygen species. Blue characters indicate known genes in tea.

Cold-induced VOCs, including linalool, MeSA, nerolidol, and geraniol, can regulate the freezing tolerance of neighboring plants via the ICE-CBF-COR pathway. Nerolidol can significantly induce CBF1 and slightly induce *CBF2* and *3* to enhance the cold tolerance of tea plants [22] (Fig. 6). Additional studies showed that plants can absorb these VOCs and convert them into glucosides, which can enhance cold stress tolerance [21, 113] (Fig. 6). The activation of the MAPK and WRKY pathways by (*E*)-nerolidol can enhance the expression of *OPR* and *PAL*, leading to an increase in the production of JA, H_2_O_2_, and ABA against insects and pathogens in tea plants (Fig. 3) [19]. Tea plants can take up airborne eugenol and use UGT71A59 to catalyze its conversion into glycosides, thereby improving the cold and drought tolerance of the plants. Under cold and drought stress, eugenol enhances ROS scavenging, regulates the expression of *CBF1*, which increases cold tolerance, and alters ABA homeostasis and stomatal closure, which improves drought tolerance in tea plants [114]. Further research is needed on what strategies plants adopt in the face of multiple stresses.

**Figure 7:**
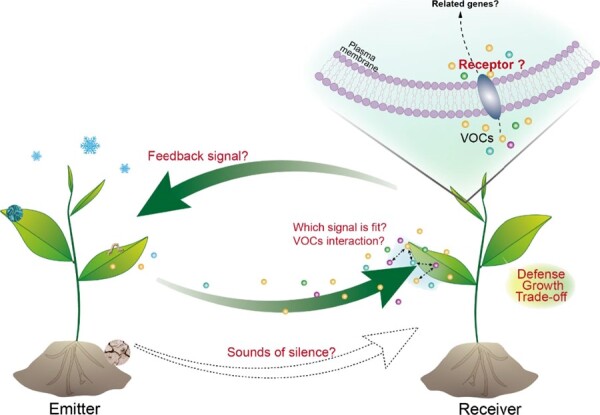
Future perspectives.

## Future perspectives

Ample evidence now suggests that a single volatile compound can serve multiple functions for plants, depending on their physiochemical characteristics. Plant volatiles display distinct spatiotemporal release and transfer patterns. Some volatiles are rapidly released from injured tissues, while others are synthesized through enzymatic cascades, leading to a slower production process [56, 115]. Certain volatiles remain stable and can disperse over considerable distances, while others are unstable and undergo rapid degradation [116].

An illustration of this can be seen with GLVs, which are rapidly released in response to stress, primarily from injured areas [117]. Consequently, they serve as a prompt but non-specific signal of ongoing damage and can be utilized by natural predators accordingly [118]. These characteristics also make GLVs prone to functioning as damage-associated molecular patterns in the regulation of plant defense [119]. In contrast, volatiles such as terpenes diffuse at a slower rate and require more time for synthesis due to specialized biosynthetic processes. As a result, they can function as highly precise signals for specific herbivores and other plants, as well as constitutive defenses or phytoanticipins. Therefore, terpenoids may be less suitable as rapid, localized defense signals [12].

Like other plants, VOCs act as chemical signals that enable communication between neighboring tea plants, aiding in the coordination of defense responses and enhancing their overall resistance. Reviewing research performed in recent years, we found that most attention was focused on insect induced VOCs; pathogen-induced and drought stress-induced volatiles are rarely studied. Besides, tea plants are rich in a wide variety of volatile compounds, but the few volatiles that have been studied are similar to those of other plants; is it that many volatiles are ignored?

While significant research has been conducted on volatile-induced plant–plant communication in tea plants, the field is still evolving. New discoveries and insights continue to deepen our understanding of the intricate mechanisms and specific compounds involved in these interactions.

VOC-mediated plant–plant communication experiments revealed that VOC signals alter the physiology and morphology of plants to modulate their growth strategies and enhance fitness. However, many outstanding questions remain to be solved.

Recent reviews have put forth mechanisms for the perception and signaling of VOCs. These volatile compounds can enter leaves either through stomata or by penetrating the cuticular wax layer. Subsequently, VOCs can be detected by receptor/receptor complexes, leading to cellular responses or uptake for subsequent metabolic processing [120, 121]. Some studies suggest that plants may have odorant-binding proteins (OBPs) to transport VOCs to olfactory receptors, which activate the transduction pathway for VOC perception. The TOPLESS protein can specifically bind β-caryophyllene [88]. Additionally, the COI1 protein, when assembled with a member of the ZIM-domain JAZ protein family (COI1-JAZ), acts as a high-affinity receptor protein for bioactive JA [122]. These studies suggest that there are protein receptors in plants that can bind and transport VOCs; however, the molecular mechanisms responsible for plant VOC perception are still not well understood, and it is not yet clear how plants sense and respond to these VOCs during interactions between plants (Fig. 7).

It is believed that the emitter benefits indirectly by conveying information about herbivory only to its own relatives, which can increase its inclusive fitness [123]. Nonetheless, this situation would require selection for the release of signals that are specific to the genotype and can only be detected, interpreted, and acted upon by related individuals. Whether the emitter benefits from warning its neighbors in nature remains unclear. No studies to date have determined whether receptor plants release some VOCs to respond to their emitters (Fig. 7).

Furthermore, plants are exposed to various individual VOC cues and signals or specific combinations of VOCs, where one or more important signals can override the effects of the other, which can cause plants to engage in inappropriate responses. In addition, the same VOCs are induced under different types of stress; for example, GLVs such as (*Z*)-3-hexenol and terpenoids such as nerolidol can be released from tea plants under low-temperature stress, insect stress, or drought stress. These substances have different functions and mechanisms of action. How do plants make sure they recognize what is really useful and respond correctly? Additional studies are needed to determine how plants cope with a diverse array of VOCs in their environment and how plants prioritize their responses to specific cues/signals (Fig. 7).

Numerous studies have offered potential mechanistic explanations for plant–plant communication by demonstrating that volatile cues can regulate various hormonal pathways, including JA, SA, and ABA signaling. Many of these pathways interconnect with one another through crosstalk. Although there have been numerous studies of crosstalk between hormones, it remains unclear how VOCs interact with one another (Fig. 7).

Moreover, in volatile-deficient environments, drought stress-induced VOCs decrease the drought tolerance of neighbors, while salt stress-induced VOCs increase the salt tolerance of neighbors. What is the function of ‘the sound of silence’ (Fig. 7)?

There is a trade-off between plant growth and defense capacity [124]. The predominant strategy for mediating this trade-off is resetting and recovery; however, there is a significant gap in our knowledge regarding the mechanisms underlying memory dissipation or ‘forgetfulness’ [125]. The effects of priming can persist between generations [126], but the magnitude of the temporal persistence of these VOC-primed memories remains unclear. Additional studies are needed to clarify how plants balance defense benefits, growth, and/or reproduction after exposure to stress-related volatiles of their neighbors (Fig. 7).

## Acknowledgements

This study was financially supported by the National Key Research and Development Program of China (2022YFF1003103), the National Natural Science Foundation of China (U22A20499 and 32022076), the Deutsche Forschungsgemeinschaft (DFG SCHW 634/34-1), and the China Postdoctoral Science Foundation 2022 M720193.

## Author contributions

J.J. and C.S. designed the review. J.J. wrote the first draft. M.Y.Z., M.T.Z, T.J., M.L., G.Y., J.W., D.G., and Y.P. contributed to different parts of the review, T.D.H. and W.S. contributed to the revision and improvement of the text.

## Data availability

All relevant data can be found within the paper.

## Conflict of interest

The authors declare that they have no conflict of interest.
